# The Correlations of Disease Activity, Socioeconomic Status, Quality of Life, and Depression/Anxiety in Chinese Patients with Systemic Lupus Erythematosus

**DOI:** 10.1155/2013/270878

**Published:** 2013-06-20

**Authors:** Biyu Shen, Wei Tan, Guijuan Feng, Yan He, Jinwei Liu, Weijun Chen, Xiaoqin Huang, Zhanyun Da, Xujuan Xu, Hong Liu, Zhifeng Gu

**Affiliations:** ^1^Department of Nursing, The Second Affiliated Hospital of Nantong University, Nantong, Jiangsu 226001, China; ^2^School of Nursing, Nantong University, Nantong, Jiangsu 226001, China; ^3^Department of Rheumatology, The Affiliated Hospital of Nantong University, Nantong, Jiangsu 226001, China

## Abstract

The prevalence of psychological problems is frequent in systemic lupus erythematosus (SLE) patients and appears to be increasing. The current study investigated the relationship among disease parameters, quality of life, and the psychological status in Chinese patients with SLE. A self-report survey design was administered to 170 SLE patients and 210 healthy individuals using the Self-Rating Anxiety Scale, the Self-Rating Depression Scale, and the Short Form 36 health survey (SF-36). Our results showed that 20.3% SLE patients had anxiety, and 32.9% had depression, which were significantly higher than the control group (7.1%, 14.3%, resp.). And there were significant correlations among socioeconomic status (SES), disease activity, and anxiety/depression in SLE patients. Meanwhile, SF-36 analysis results revealed that VT, PF, and RP scales were the most powerful predictors of anxiety of SLE patients, and SLEDAI, VT, PF, SF, and RE domains were significantly accounted for anxiety. In summary, there were significant relationships among disease parameters, quality of life, and anxiety/depression in Chinese SLE patients. Therefore, it is necessary to have psychiatric and psychological evaluations and formulate an integrated approach for managing mental health in Chinese lupus patients, especially those who have high disease activity, low SES, and poor quality of life.

## 1. Introduction

Systemic lupus erythematosus (SLE) is a chronic inflammatory autoimmune disease that may affect multiple organ systems, including the central nervous system (CNS) [1]. Psychiatric symptoms are present in the majority of patients with SLE, among which major depression is the most common psychiatric manifestations [2]. Depression presented in 11%–39% of patients may be the initial symptom before the diagnosis of SLE [3]. It was reported that there were 4 times higher prevalence of depression in SLE compared to a matched, non-SLE population. In addition, anxiety is quite common in SLE patients, often as a reaction to the illness. Ainiala and colleagues have reported that the anxiety disorders were twice as prevalent among SLE patients as compared to the controls [4]. 

Even though SLE presents accompany with a wide variety of treatable psychiatric symptoms, such as depression and anxiety, they rarely seek and receive adequate treatment [5]. Overlooking anxiety and depression may have severe consequences for the patients, such as increased incidence of cardiovascular disease [6], myocardial infarction [7], suicidal ideation [8, 9] and death [10], decreased quality of life [11, 12], disability, and the loss of employment. Anyone, in turn, can worsen anxiety and depression symptoms [5]. 

The pathogenesis of psychiatric symptoms in lupus is still not well understood, but in which genetic and environmental factors may play a pivotal role. Depression and anxiety may also be present as a reaction to a serious recurring, painful illness, which is associated with visible symptoms such as insomnia, fatigue, and limited functioning [13, 14]. Socioeconomic status (SES) is broadly employed in health research, signaling the importance of socioeconomic factors for health outcomes. Low SES is generally associated with high psychiatric morbidity, depression [15], and mortality [16]. Whether depression and anxiety are associated with lupus activity remains debatable.

There are several studies focus on psychological problems in China lupus patients. A study from Hong Kong has found that anxiety disorder was present in 22% SLE patients, and 18.2% had depression [17]. A study from Anhui medical university has reported that the prevalence of depression was 59.3% and correlated with suicidal ideation in SLE patients [8]. But there are few studies that focus on disease parameters, quality of life, and depression/anxiety in SLE patients from China mainland.

Thus, the aim of this study was to examine the relationship among disease parameters, quality of life, and the psychological status in Chinese patients with SLE. Moreover, we wished to ascertain the possible risks of anxiety and depression.

## 2. Patients and Methods

### 2.1. Participants

SLE patients were recruited from Affiliated Hospital of Nantong University between January 2010 and July 2011. A total of 170 SLE patients and 210 healthy individuals were consecutively invited to participate in a single-center cross-sectional study. Healthy individuals were used as the control group. All patients fulfilled the 1997 American College of Rheumatology (ACR) revised criteria for the classification of SLE. Patients were excluded based on the following: (1) they did not complete questionnaire; (2) they had comorbidities (e.g., serious infections or cardiac, respiratory, gastrointestinal, neurological, or endocrine diseases) that could influence SLE activity. Control subjects were excluded if they exhibited current or history of other systemic diseases or psychiatric disorders. This study was approved by the Ethics Committee of Affiliated Hospital of Nantong University, and written informed consent was obtained from all participants.

### 2.2. Measures of Clinical Variables

The Systemic Lupus Erythematosus Disease Activity Index (SLEDAI) was used to measure disease activity [18].

### 2.3. The Revised Self-Rating Anxiety Scale (SAS) [19]

SAS was used to evaluate the level of anxiety-related symptoms during the week prior to the survey. This self-administered test has 20 questions, with 15 items reflecting increasing anxiety levels and 5 questions reflecting decreasing anxiety levels. Each question was scored on a scale from 1 to 4 (rarely, sometimes, frequently, and always). The scores ranged between 20 and 80: scores greater than 70 suggest severe anxious symptoms, scores between 60 and 69 indicate moderate to marked anxiety, scores between 50 and 59 suggest minimal to mild anxiety, and scores less than 50 indicate no anxious symptoms.

### 2.4. The Revised Self-Rating Depression Scale (SDS) [20]

SDS is a 20-item questionnaire designed to assess mood symptoms over the past week (e.g., “I feel downhearted, blue and sad”). Each item is scored on a Likert scale ranging from 1 to 4; scores greater than 70 suggest severe depressive symptoms, scores between 60 and 69 indicate moderate to marked depression, scores between 50 and 59 suggest minimal to mild depression, and scores less than 50 indicate no depressive symptoms.

### 2.5. Measure of the Quality of Life [21]

The patient's general health status was measured using the Short Form- (SF-) 36 questionnaire, which measured eight multi-item dimensions: physical functioning (PF, 10 items); role limitations due to physical problems (RP, four items); role limitations due to emotional problems (RE, three items); social functioning (SF, two items); mental health (MH, five items); energy/vitality (VT, four items); body pain (BP, two items); and general health perception (GH, five items). For each dimension, item scores were coded, summed, and transformed on a scale from 0 (worst possible health state measured by the questionnaire) to 100 (best possible health state).

### 2.6. Statistical Analysis

The data were expressed as means ± SDs for continuous variables and as frequencies (%) for categorical variables. The Statistical Package for SPSS 18.0 was used for all data management and analyses. Descriptive analyses were performed to investigate the participants' characteristics. Student's *t*-test was used in independent groups for parametric variables, and the Spearman's correlation analysis was used to assess the correlation between parametric variables. Stepwise regression analyses were conducted for SAS and SDS scores separately to explore the significant predictors of dimorphic concerns. We considered *P* < 0.01 and *P* < 0.001 to be highly statistically significant and *P* < 0.05 to be statistically significant.

## 3. Results

### 3.1. Sample Characteristics

12 SLE patients and 14 healthy individuals did not complete the questionnaire, resulting in the enrollment of 158 SLE patients (14 males and 144 females) and 196 healthy individuals (20 males and 176 females) in the current study.  Table 1 showed their demographic data, medical and psychological variables. There was no significant difference in the ages, genders, marital status, education, work status, income/person, and menstrual history between the SLE patients and the controls. The SAS and SDS scores were significantly higher in the SLE group compared to the control group. According to the cut-off scores, anxiety disorder was present in 32/158 (20.3%), and 52/158 (32.9%) had depression, which were significantly higher than the healthy group ((14/196, 7.1%) and (28/196, 14.3%), resp.) (*P* < 0.01). As shown in Table 2, the scores of all the 8 scales were lower in SLE patients compared with healthy individuals. There was significant difference between physical functioning (PF), role limitations due to physical problems (RP), role limitations due to emotional problems (RE), mental health (MH), and energy/vitality (VT) in SLE and control group (*P* < 0.05).

### 3.2. Correlations between Psychological Scores, Disease Parameters, and Quality of Life in SLE Patients

Previous studies have shown that low socioeconomic factors (SES) were generally associated with high psychiatric morbidity, depression, and anxiety [22]. As show in Table 3, we have found that there were significant correlations between SES (low education, work status, and income) and anxiety/depression in SLE patients. In addition, gender and menstrual history were some examples of depression risk factors. There was a significant positive correlation between anxiety/depression severity (assessed using SAS/SDS score) and disease activity (SLEDAI score). Previous studies have found that impaired quality of life and functional disability were independent risk factors of psychological disorders [12]. In the present study, we found that all the 8 scales of SF-36 domains and PCS/MCS were significantly correlated with SAS and SDS scores except body pain (BP) scale (*P* < 0.05).

### 3.3. Stepwise Regression Analysis for Anxiety and Depression

Multiple stepwise regression analysis revealed that VT, PF, and RP scales of SF-36 were the most powerful predictors of anxiety of SLE patients (Table 4). Meanwhile, SLEDAI, VT, PF, SF, and RE domains of SF-36 were significantly accounted for anxiety (Table 5).

## 4. Discussion

The present study confirmed that Chinese SLE patients were more likely to suffer from anxiety and depression than healthy individuals. Psychological problems significantly correlated with SES, disease status, and quality of life. SLE patients with anxiety and depression were in low SES and had worse disease status, lower quality of life. Among the assessed parameters, VT, PF, and RP scales of SF-36 were major contributors to anxiety in SLE patients, while disease activity and VT, PF, SF, and RE domains of SF-36 contributed to depression.

SES is broadly employed in health research, signaling the importance of socioeconomic factors for health outcomes. Previous study has found that poorer coping styles, ongoing life events, stress exposure, and weaker social support were some examples of depression risk factors that were more prevalent in lower SES groups [23]. Regarding the direction of the association for SES and depression, results more consistently supported the idea that causation (low SES increases risk of depression) outweighed selection (depression hinders social mobility), although both directions may operate simultaneously [17]. It is well known that SES is multifactor. Occupation [5, 24, 25], education, and income [26] were frequently used as measures of SES. With notable exceptions, there were significant relationships between anxiety/depression and SES [5]. There was a substantial body of research linking SES, anxiety/depression, and SLE. Waheed et al. found that educational qualification had significant association with anxiety and depression. Marital status, gender, economic activity, and monthly family income had no effect on the frequency of anxiety and depression [27]. In the present study, we have found that SLE patients who had low education, unemployed, and low income were prone to anxiety/depression. Female gender and younger age have well-known associations with depression and confound the SES anxiety/depression relationship in SLE [28]. In the present study, gender was independently associated with depression in SLE. Interestingly, we have found that there were significant correlation between abnormal menstrual history and depression. Whether anxiety/depression is associated with lupus activity remains debatable. Walker SE and colleagues reported that the anxiety severity did correlate positively with SLE activity [29]. Nery et al. reported a significant positive correlation between depression and disease activity [30]. In contrast, other studies reported that there was not relationship between lupus activity and presence of a major depressive episode [31]. In the present study, our group found significant positive correlation between anxiety/depression and disease activity. Health-related quality of life in lupus was found to be significantly worse in comparison with control group. Recent study reported that SLE patients who had significantly poorer health-related quality of life were significantly more depressed and anxious than their healthy counterparts [11]. We have also found the PCS and MCS and all 8 domains of SF-36 except BP were significantly worse than control subjects. The results were similar with the study from Hong Kong. We have also found that the SLEDAI scores were a strong predictor of depression in patients with SLE.

 Notably, the results of the present study demonstrated that anxiety in Chinese SLE patients differs from SLE patients in other countries. This could be explained by some cultural features which may influence mental disease diagnosis and management in China. We have found that the prevalence of depression was higher than Hong Kong, and it might be due to cultural differences and SES such as income and medical insurances policy. 

In order to identify which variables were most significantly correlated with anxiety and depression, stepwise regression analysis was used. We have found that VT, PF, and RP scales of SF-36 were the most powerful predictors of anxiety of SLE patients. Meanwhile, SLEDAI, VT, PF, SF, and RE domains of SF-36 were significantly accounted for anxiety. It could be explained that impaired quality of life and functional disability were independent risk factors for psychological disorders. 

 A possible limitation of the present study was that all patients involved in the survey were from only one center and its failure to differentiate between men and women; the gender differences in SLE patients require further analysis in a future study. Another limitation of the study was that we did not detect the impact of proinflammatory cytokines on depression. Recent study reported that higher serum TNF-*α* level was independently associated with poorer health-related quality of life and more severe depressive symptoms in SLE patients in Singapore [11]. 

 In summary, our study indicated that psychological problems were frequent in Chinese SLE patients. Severe disease status and reduced quality of life significantly correlated with anxiety and depression. Disease activity was higher in anxious and depressed subgroups. Quality of life was decreased in depressed subgroups. Impaired mental health and pain were the most powerful predictors of anxiety and depression. Low SES was independently associated with poor mental health. These findings confirmed the importance of psychosocial interventions in combination with medical therapy for SLE patients.

## Figures and Tables

**Table 1:** Demographic, psychological, and disease characteristics in SLE patients and controls.

Variables	SLE patients (*N* = 158)	Control subjects (*N* = 196)	*P*
Female gender^a^	144 (91.2)	176 (89.8)	0.76
Age, years^b^	32.9 ± 10.2	35.0 ± 11.4	0.20
SAS (≥50)^a^	32 (20.3)	14 (7.1)	<0.01
SDS (≥53)^a^	52 (32.9)	28 (14.3)	0.003
SLEDAI	11.8 ± 9.5		
Marital status^b^			
Single	32 (20.3)	56 (18.6)	0.20
Married	126 (79.7)	140 (71.4)	
Education^b^			
<9 years	86 (54.4)	96 (49.0)	0.47
≥9 years	72 (45.6)	100 (51.0)	
Work status^b^			
Working	30 (19.0)	44 (22.5)	0.57
Unemployed	128 (81.0)	152 (77.5)	
Income/person^b^			
≤2000 yuan	100 (63.3)	118 (60.2)	0.68
>2000 yuan	58 (36.7)	78 (39.8)	
Menstrual history^b^			
Normal	96 (66.7)	102 (58.0)	0.26
Abnormal	48 (33.3)	74 (42.0)	

^a^Mean ± SD. ^b^Number (percentage).

SAS: revised Self-Rating Anxiety Scale; SDS: revised Self-Rating Depression Scale; SLEDAI: Systemic Lupus Erythematosus Disease Activity Index.

**Table 2:** Correlations between psychological scores, disease parameters, and quality of life in SLE patients.

Variables	SAS		SDS	
	*r*	*P*	*r*	*P*
Domains of SF-36				
PCS	−0.53	<0.0001	−0.53	<0.0001
MCS	−0.68	<0.0001	−0.73	<0.0001
PF	−0.49	<0.0001	−0.54	<0.0001
RP	−0.55	<0.0001	−0.52	<0.0001
BP	−0.05	0.66	0.05	0.65
GH	−0.36	0.001	−0.37	0.0009
VT	−0.4	0.0003	−0.43	0.0001
SF	−0.49	<0.0001	−0.57	<0.0001
RE	−0.63	<0.0001	−0.64	<0.0001
MH	−0.31	0.005	0.34	0.003

SAS: revised Self-Rating Anxiety Scale; SDS: revised Self-Rating Depression Scale; PCS: physical components summary; MCS: mental components summary; PF: physical functioning; RP: role limitations due to physical problems; RE: role limitations due to emotional problems; SF: social functioning; MH: mental health; VT: energy/vitality; BP: body pain; GH: general health perception.

**Table 3:** Disease status and quality of life in the anxious and depressed subgroups.

Variables	SAS			SDS		
	<50	≥50	*P*	<53	≥53	*P*
Age^a^	32.3 ± 10.3	35.2 ± 9.6	0.32	32.4 ± 10.6	34.0 ± 9.4	0.51
Sex^b^						
Male	6 (9.5)	1 (6.3)	0.68	7 (13.2)	0 (0.0)	0.05
Female	57 (90.5)	15 (92.7)		46 (86.8)	26 (100.0)	
BMI	21.2 ± 2.7	21.1 ± 2.9	0.93	21.1 ± 2.8	21.4 ± 2.6	0.61
Marital status^b^						
Single	15 (23.8)	1 (6.3)	0.25	14 (26.4)	2 (7.7)	0.11
Married	48 (76.2)	15 (93.7)		38 (73.6)	24 (92.3)	
Education^b^						
<9 years	29 (46.0)	14 (87.5)	0.003	23 (43.4)	20 (76.9)	0.005
≥9 years	34 (54.0)	2 (12.5)		30 (56.6)	6 (23.1)	
Work status^b^						
Working	15 (23.8)	0 (0)	0.03	13 (24.5)	2 (7.7)	0.07
Unemployed	48 (76.2)	16 (100.0)		40 (75.5)	24 (92.3)	
Income/person^b^						
≤2000 yuan	36 (57.1)	14 (87.5)	0.02	28 (52.8)	22 (84.6)	0.006
>2000 yuan	27 (42.9)	2 (12.5)		25 (47.2)	4 (15.4)	
Menstrual history^b^						
Normal	39 (68.4)	9 (60.0)	0.54	36 (79.2)	12 (46.2)	0.006
Abnormal	18 (31.6)	6 (40.0)		10 (20.8)	14 (53.8)	
Years since diagnosis of SLE^b^						
<1	11 (17.5)	2 (12.5)	0.85	10 (18.9)	3 (11.5)	0.63
1–5	31 (49.2)	9 (56.3)		27 (50.9)	13 (50.0)	
>5	21 (33.3)	5 (31.2)		16 (30.2)	10 (38.5)	
SLEDAI^a^	10.4 ± 7.3	17.1 ± 14.7	0.01	10.3 ± 7.7	14.8 ± 12.1	0.046
Domains of SF-36						
PCS^a^	256.7 ± 60.7	158.1 ± 51.7	<0.0001	264.0 ± 58.6	181.1 ± 61.5	<0.0001
MCS^a^	282.8 ± 67.0	161.7 ± 60.1	<0.0001	295.1 ± 56.9	183.3 ± 72.9	<0.0001
PF^a^	86.8 ± 14.1	61.3 ± 28.8	<0.0001	88.9 ± 12.2	66.9 ± 26.1	<0.0001
RP^a^	52.4 ± 42.5	4.7 ± 13.6	0.0001	57.1 ± 43.1	13.5 ± 23.7	<0.0001
BP^a^	63.8 ± 26.8	54.0 ± 20.0	0.18	63.6 ± 26.6	58.2 ± 24.4	0.38
GH^a^	53.6 ± 14.0	38.1 ± 13.3	0.0001	54.4 ± 12.6	42.5 ± 16.8	0.0007
VT^a^	66.6 ± 16.1	48.1 ± 19.5	0.0002	68.8 ± 15.0	50.8 ± 18.7	<0.0001
SF^a^	86.7 ± 29.3	64.1 ± 34.4	0.01	91.7 ± 26.8	62.5 ± 31.6	0.0001
RE^a^	67.2 ± 38.1	0 ± 0	<0.0001	71.7 ± 36.0	16.7 ± 33.0	<0.0001
MH^a^	62.3 ± 15.1	49.5 ± 19.4	0.006	62.9 ± 13.9	53.4 ± 20.3	0.017

^a^Mean ± SD. ^b^Number (percentage).

SAS: revised Self-Rating Anxiety Scale; SDS: revised Self-Rating Depression Scale; PCS: physical components summary; MCS: mental components summary; PF: physical functioning; RP: role limitations due to physical problems; RE: role limitations due to emotional problems; SF: social functioning; MH: mental health; VT: energy/vitality; BP: body pain; GH: general health perception.

**Table 4:** Stepwise regression analyses of medical and psychological variables and their relationship to SAS in SLE patients.

SAS	Coef.	Std. Err.	*t*	*P*	[95% CI]
VT	−0.15	0.05	−3.00	0.004	−0.25, −0.05
PF	−0.11	0.05	−2.27	0.026	−0.22, −0.01
RP	−0.09	0.02	−3.64	<0.001	−0.14, −0.04

SAS: revised Self-Rating Anxiety Scale; VT: energy/vitality; PF: physical functioning; RP: role limitations due to physical problems.

**Table 5:** Stepwise regression analyses of medical and psychological variables and their relationship to SDS in SLE patients.

SDS	Coef.	Std. Err.	*t*	*P*	[95% CI]
SLEDAI	0.19	0.09	2.08	0.04	0.01, 0.37
VT	−0.19	0.05	−3.48	0.001	−0.30, −0.08
PF	−0.13	0.05	−2.60	0.01	−23.8, −0.03
SF	−0.09	0.03	−2.69	0.009	−0.15, −0.02
RE	−0.08	0.02	−3.13	0.003	−0.12, −0.03

SDS: revised Self-Rating Depression Scale; SLEDAI: Systemic Lupus Erythematosus Disease Activity Index; VT: energy/vitality; PF: physical functioning; SF: social functioning; RE: role limitations due to emotional problems.
